# Multiomic analysis reveals that the flavonoid biosynthesis pathway is associated with cold tolerance in *Heracleum moellendorffii* Hance

**DOI:** 10.3389/fpls.2025.1544898

**Published:** 2025-03-14

**Authors:** Guan Liu, Huan Gao, Yu Song, Hanhui Wang, Dongye Zhang, Yang Wang, Shuo Liu, Zhonghua Li, Changhua Liu, Yan Sun

**Affiliations:** ^1^ College of Advanced Agriculture and Ecological Environment, Heilongjiang University, Harbin, China; ^2^ State Key Laboratory of Tree Genetics and Breeding, College of Forestry, Northeast Forestry University, Harbin, China; ^3^ Heilongjiang Greater Hinggan Mountains Region Agriculture Forestry Research Institute, Da Hinggan Ling, China

**Keywords:** *Heracleum moellendorffii* Hance, cold stress, multi-omics, flavonoid biosynthesis, transcription factor

## Abstract

*Heracleum moellendorffii* Hance is a perennial herbaceous plant that is adaptable to cold environments and has both edible and medicinal value. Given that no reference genome for this species is available, we constructed a high-quality transcript isoform library using full-length transcriptome sequencing and conducted a comparative genomic analysis. Samples were obtained from plants that had been subjected to cold stress for 12, 24 and 36 hours (Cold_12, Cold_24, and Cold_36, respectively) and from control plants (Cold_0) that were not subjected to cold stress and used in transcriptome and nontargeted metabolome analyses. Compared with the genes expressed in CK (Cold_0), the number of differentially expressed genes (DEGs) in Cold 12, Cold_24, and Cold_36 increased gradually over time; plants subjected to 12, 24 and 36 hours of cold stress displayed 669, 6084, and 24,129 DEGs, respectively. The DEGs were clustered into 8 subclasses by k-means clustering; subclasses 2, 3, 4, and 7 were enriched in pathways related to “flavonoid biosynthesis”. Nontargeted metabolome analysis revealed that 3719 annotated metabolites were shared by all four groups of samples. We identified 1186, 1087, and 1097 differentially accumulated metabolites (DAMs) in three comparisons: Cold_12 vs. CK, Cold_24 vs. CK, and Cold_36 vs. CK, respectively. The DAMs were predominantly enriched in the “flavonoid biosynthesis pathway”. Through WGCNA, we obtained five modules and 29 flavonoid-related metabolites with extremely significant module−metabolite paired relationships (|correlation coefficient|> 0.9, *P* < 0.01). We analysed the DEGs and DAMs of the flavonoid biosynthetic pathway in *H. moellendorffii* Hance under cold stress and constructed a correlation network between transcription factors (TFs) and structural genes in the pathway. RT−qPCR was used to confirm the expression of four hub genes from the WGCNA, six TFs, and 15 structural genes of the flavonoid biosynthetic pathway. These data provide a foundation for functional genomics studies of *H. moellendorffii* Hance and contribute to the study of the molecular mechanisms and transcriptional regulation of flavonoid accumulation by TFs under cold stress conditions in plants.

## 1 Introduction

*Heracleum moellendorffii* Hance, a perennial herbaceous geophyte belonging to the family Apiaceae and the genus *Heracleum*, is representative of Northeast China’s mountain wild vegetables that possess both nutritional and economic value (Li et al., 2019). Although it belongs to the same family as the common culinary vegetable *Apium graveolens* L., *H. moellendorffii* presents a rich nutritional profile in multiple vitamin metabolites, amino acids, mineral substance, as well as flavonoids, coumarins, and saponins (Li et al., 1997; Wang et al., 2017). Thus, *H. moellendorffii* has medicinal and health benefits. Its aerial parts are often used as a source of medications for diabetes, and its roots, which have effects similar to those of *Angelica dahurica*, are commonly used in traditional Chinese medicine. It can be used to treat rheumatism and hypertension and has anti-fatigue, analgesic and anti-inflammatory effects (Liu et al., 2019a; Kim H, et al., 2019). *H. moellendorffii* thrives in environments with high organic matter content, deep soil, abundant groundwater, and dry, loose surfaces and is widely adaptable and susceptible to few pests and diseases. Its strong cold tolerance allows it to survive several hours at -4°C without damage (Li et al., 2019).

Cold stress is one of the most common abiotic stresses. When plants are subjected to cold stress, changes in cell morphology, subcellular structure, and protoplasm occur and are accompanied by disordered respiration, altered enzyme activities, and metabolic imbalances (Chinnusamy et al., 2007; Mahajan and Tuteja, 2015). Earlier studies of the cold stress response focused on physiological aspects such as morphological changes and damage, changes in the concentrations of metabolic products, and the activity of protective enzymes. In recent years, with the commercialization of various omics platforms such as high-throughput sequencing, gas chromatography−mass spectrometry (GC−MS), and liquid chromatography−mass spectrometry (LC−MS), multiomics approaches for dissecting the molecular mechanisms of plant cold stress have become increasingly prevalent (Cheong et al., 2020; Dong et al., 2020; Lee et al., 2020). These studies typically involve cold stress treatment of biological materials, sampling at various time points, and identification of differentially expressed genes (DEGs) through transcriptome sequencing, followed by clustering of transcription factors (TFs) within the DEGs and GO and KEGG enrichment analysis. These studies provide a landscape of the entire transcriptome and DEGs, and the mechanisms through which organisms respond to cold stress are also described from the perspective of the gene families that are potentially involved in stress resistance (such as CBF, WRKY, and MYB) and the GO and KEGG categories related to stress resistance. Related studies have been conducted on crops such as rice (Pan et al., 2020), corn (Frey et al., 2020), wheat (Kruse et al., 2020), and peanuts (Bonthala et al., 2016). There are also reports on the molecular functions of particular DEGs, such as the ability of ShNAC1 overexpression in tomato plants to reduce cold tolerance; further research suggests that this transcription factor may be involved in ethylene biosynthesis and signal transduction pathways (Wang et al., 2024). Similarly, the cold-induced TF SlGRAS4 in tomato enhances cold stress tolerance by binding to the promoter region of SICBF and activating its expression without inhibiting normal plant growth (Liu et al., 2020). Studies of this type often focus on specific TFs involved in cold stress, such as MYB (An et al., 2020), DREB (Zhang et al., 2020), ICE1 (Tang et al., 2020), WRKY (Zhang et al., 2016), NAC (An et al., 2018), and bZIP (Liu et al., 2019b).

Metabolites are the ultimate manifestation of the transcription of genes into mRNAs, which are further translated into proteins, and they also extensively participate in the response of plants exposed to cold stress. In plants, cold stress may lead to the accumulation of toxic products due to metabolic imbalances. Soluble sugars act as important signalling molecules during cold stress (Rolland et al., 2006). Lipid content changes significantly when plants are exposed to cold stress (Cheong et al., 2020). Cold stress also induces the accumulation of amino acids and carbohydrates (Kou et al., 2018). Other metabolites, such as flavonoids (Song et al., 2024) and polyamines (Kovács et al., 2010), are also involved in the adaptation of plants to cold stress.

In this work, we focused on the involvement of flavonoids in the regulation of cold stress. Flavonoid biosynthesis is an important branch of the phenylpropanoid metabolic pathway and plays a crucial role in plant growth, development, and stress resistance (Daryanavard et al., 2023). Cold temperatures have been reported to activate the phenylpropanoid pathway, leading to the accumulation of flavonoid compounds and thereby reducing oxidative damage in *Poa crymophila* Keng (Wang et al., 2021) and bell pepper (Xu et al., 2023). Sun et al. (2021) reported that the chalcone isomerase gene (CHI) is specifically upregulated in cold-tolerant kiwifruit varieties exposed to cold stress, and the flavonoid metabolic pathway is also specifically upregulated, increasing the ability of these plants to scavenge reactive oxygen species (ROS). Similarly, chalcone and stilbene synthases, which participate in the flavonoid biosynthetic pathway, are involved in the cold stress response in tobacco (Hu et al., 2022). Studies of this type often focus on differences in the expression of structural genes and flavonoid-related metabolites in the flavonoid metabolic pathway. In addition, it has been reported that TFs play a role in regulating flavonoid biosynthesis. For example, MYB transcription factors constitute an important class of regulators of the production of flavonoid metabolites. R2R3-MYB transcription factors often act as transcriptional activators or repressors to directly or indirectly regulate the expression of structural genes related to flavonoid biosynthesis (Zhao et al., 2013). The MBW (MYB-bHLH-WD40) complex is also involved in regulation (Xu et al., 2015). Other transcription factors, such as NAC, ZIP, and WRKY, have also been reported to participate in the regulation of the flavonoid biosynthesis pathway (Morishita et al., 2009; Malacarne et al., 2016; Zhang et al., 2023).

With the development of sequencing technology, genome assembly, an important foundation for functional genomics in the postgenomic era, has become increasingly common. The reference genome of celery, which also belongs to the family Apiaceae, has been released (Li et al., 2020; Song et al., 2021). Fundamental research on *H. moellendorffii* is relatively rare; there are only a few reports involving the use of omics methods to study seed dormancy characteristics and disease resistance (Li et al., 2019; Liu et al., 2019a, 2024b). The absence of a high-quality reference genome severely hinders the development of a functional genome that can be used in molecular breeding of *H. moellendorffii* and the in-depth development and utilization of its resources.

*H. moellendorffii* grows normally in low-temperature environments and adapts well to cold. This study is the first to construct a full-length transcriptome library of *H. moellendorffii* and thereby provide a reference for transcriptomics and other studies. Through time-course transcriptome and metabolome analyses, structural genes and regulatory factors of the flavonoid pathway that respond to exposure of the plant to low temperatures were subsequently identified. This research may lay a molecular foundation for subsequent genetic improvement and benefit the protection of germplasm resources of characteristic vegetables in the Apiaceae family, and the development and utilization of specialty crops.

## 2 Materials and methods

### 2.1 Plant material source and sampling

Seeds of *H. moellendorffii* were collected from the wild, cleaned, and air dried for later use. Subsequently, seeds with plumped grains were washed with clean water, disinfected with 75% ethanol for 30 seconds, and soaked in distilled water for 24 hours. After incubation at low temperatures to break dormancy, the seeds of *H. moellendorffii* were sown in pots within the greenhouse at the Horticultural Engineering Center of Northeast China Agricultural University (126°67′ latitude, 45°73′ longitude) in November 2021. For PacBio full-length sequencing, plant material was collected in 2024 from experimental fields in a greenhouse. We selected the roots, stems, leaves, flowers, and 7-day postpollination fruits of two-year-old *H. moellendorffii*. RNA was extracted from approximately 0.3–0.5 g of each tissue sample and used to construct a full-length transcriptome reference library. When the seedlings had developed two fully expanded true leaves, they were subjected to low-temperature stress at 4°C, which was set empirically (Guy et al., 2008; Jan et al., 2024). Leaf samples were collected after 0 h, 12 h, 24 h, and 36 h of low-temperature stress; these samples were designated CK (0 h), Cold_12 (12 h), Cold_24 (24 h) and Cold_36 (36 h), respectively. The samples were rapidly frozen in liquid nitrogen and stored at -80°C for use in future experiments. For transcriptome sampling, three biological replicates (approximately 0.5 g each) were obtained. For nontargeted metabolomics, six biological replicates were sampled; each sample weighed 0.3–0.5 g. Notably, three of the six metabolomic biological replicates were derived the same source of the RNA-seq samples, and the rest three were obtained from additional separate samples.

### 2.2 PacBio-based full-length transcriptome sequencing and data analysis

Total RNA was extracted from the root, stem, leaf, flower, and fruit of *H. moellendorffii*. The concentration and purity of the RNA were measured using a Nanodrop 2000 spectrophotometer. The integrity of the RNA was confirmed by agarose gel electrophoresis, and the RNA integrity number (RIN) was determined on an Agilent 2100 bioanalyzer, ensuring that all the samples had RIN values ≥ 8. RNA samples that met the quality criteria were pooled in equal amounts and used to construct full-length transcriptome sequencing libraries. The final libraries each contained ≥5 µg total RNA at ≥300 ng/μL and had OD260/280 ratios ranging from 1.8 to 2.2. Library construction was carried out using the SMARTer™ PCR cDNA Synthesis Kit (Pacific Biosciences, USA), with the following steps: 1) synthesis of full-length cDNA from mRNA via the SMARTer™ PCR cDNA Synthesis Kit (Pacific Biosciences, USA); 2) PCR amplification of the synthesized cDNA; 3) purification of the amplified full-length cDNA using PB magnetic beads; 4) end-repair of the purified full-length cDNA; 5) ligation of SMRT bell-shaped adapters to the repaired cDNA; 6) nuclease exonuclease digestion to prepare the cDNA for sequencing; and 7) further purification on PB magnetic beads to obtain the final sequencing library. Sequencing of the libraries to generate long-read sequences for transcriptome analysis was performed on the PacBio Sequel II platform.

The raw sequencing data were subjected to quality control. Sequences obtained from polymerase read fragments shorter than 50 base pairs (bp) or with accuracies less than 0.90 were discarded. The adapter sequences were cleaved and filtered out to obtain subreads. Subreads shorter than 50 bp were filtered out, resulting in clean data. Circular consensus sequences (CCS) were extracted from the original sequences on the basis of the criteria of ≥3 full passes and sequence accuracy greater than 0.90, followed by statistical analysis and assessment of the data. Full-length consensus sequences were obtained via Iso-Seq3 in PacBio SMRT Analysis software. The analysis process consisted of three main stages: full-length sequence identification, isoform-level clustering to obtain consensus sequences, and polishing of the consensus sequences. The detailed steps were as follows: 1) the reads of insert (ROI) sequences were extracted from the original raw sequences, cDNA primers and polyA were filtered out, and the sequences were categorized into full-length and non-full-length sequences and chimeric and nonchimeric sequences on the basis of the presence of 3’ primers, 5’ primers, and poly A; 2) clustering of full-length sequences from the same isoforms was performed using the iterative isoform-clustering (ICE) algorithm, which forms clusters of similar sequences, with each cluster yielding a single consensus sequence; 3) clustering of non-full-length sequences was performed using the Quiver algorithm, followed by polishing of the consensus sequences to further select high-quality sequences. The Cogent software package was used to reconstruct “fake contigs”, and the consensus sequences obtained were aligned with the reconstructed “fake contigs” using pbmm2 v1.2.1 (https://github.com/PacificBiosciences/pbmm2) with default parameter settings. The collapse tool from the Iso-Seq3 pipeline was used to remove redundancy from the alignment results, ultimately yielding a reference transcriptome library for *H. moellendorffii*. The completeness of the final transcripts was assessed via BUSCO (eukaryota_odb10) (Simão et al., 2015). The full-length transcripts were functionally annotated using five major databases (NR, UniProt, GO, KEGG, and Pfam), and the transcription factors were annotated using the Plant Transcription Factor Database (PlantTFDB) (Jin et al., 2017). MISA was used to call simple sequence repeats (SSRs) (https://webblast.ipk-gatersleben.de/misa/). LncRNAs were predicted using the CPC, CNCI, PLEK and Pfam databases (Chang et al., 2021).

### 2.3 Comparative genome analysis

Protein coding prediction to identify protein isoforms was performed using TransDecoder v.5.7.1 (https://github.com/TransDecoder/TransDecoder). The longest protein sequence for each gene was selected for further analysis. We selected eight species from the same family (umbelliferae) or genus to *H. moellendorffii* for comparative genomic analysis, and *Solanum lycopersicum* was set as an outgroup. Orthofinder v2.5.5 software (Emms and Steven, 2019) was employed to classify protein sequences from the ten species into families, and the PANTHER-18.0 database (https://pantherdb.org) was utilized to annotate the identified gene families. Gene Ontology (GO) and KEGG enrichment analyses were subsequently conducted for gene families specific to *H. moellendorffii*. All single-copy orthologous genes were extracted and used to construct a phylogenetic tree. Specifically, MAFFT v7.520 (Katoh et al., 2009) was used to align single-copy orthologue gene sequences with parameters set to –localpair –maxiterate 1000. Gblocks v0.91b (Talavera and Jose, 2007) was then used to trim the sequences (parameters: -b5=h). The concatenated sequences of all the aligned and trimmed sequences were assembled into a supergene. The best-fit model (JTT+F+R4) was determined using the ModelFinder tool within IQ-TREE (Nguyen et al., 2015). Finally, the maximum likelihood (ML) method was employed to construct the evolutionary tree using the optimal model, with bootstrap=1000 and *S. lycopersicum* designated as the outgroup. Divergence time estimation was performed using the MCMCTREE module in PAML v4.10 (Yang, 2007), with fossil calibration times obtained from the TimeTree website (http://www.timetree.org/). The contraction and expansion of gene families relative to the ancestor in *H. moellendorffii* were predicted using CAFE v5.1 (Han et al., 2013).

### 2.4 Illumina-based transcriptome sequencing and gene expression analysis

Total RNA was extracted from leaf tissue samples collected at 0 h, 12 h, 24 h, and 36 h following the beginning of exposure to cold stress. The RNA quality control methods used were the same as those used for the full-length transcriptome. High-quality RNA samples were treated with fragmentation buffer, and the mRNAs were randomly fragmented into fragments of approximately 300 base pairs (bp) using a Covaris Sonicator (Covaris, USA). Under the action of reverse transcriptase and with the aid of random primers, first-strand cDNA was synthesized using mRNA as a template, followed by synthesis of the complementary strand. The next step involved the addition of End Repair Mix (Illumina, San Diego, CA) to create blunt ends from the sticky ends, followed by the addition of an A base at the 3’ end to facilitate the ligation of adapter sequences. The products obtained after adapter ligation were purified and size selected. The selected products were subjected to PCR amplification and further purified to obtain the final sequencing library. After quantification using Qubit 4.0, the library was sequenced on the NovaSeq X Plus platform.

The raw sequencing data were processed using fastp to obtain clean data (Chen et al., 2018), with data filtering criteria consistent with those of Liu et al. (2024a). The clean data were then aligned to the full-length transcriptome reference using HISAT2 (Kim D, et al., 2019) to generate mapped data (reads) for subsequent transcript assembly and quantification of gene expression. RSEM (Li and Dewey, 2011) was used to perform TPM (transcripts per million) quantification analysis for both genes and transcripts. Genes whose TPM expression was less than 1 were filtered out before PCA (Marini and Binder, 2019) and differential gene expression analysis were conducted via DESeq2 (Love et al., 2014). The criteria for selecting differentially expressed genes (DEGs) were FDR < 0.05 and |log2FC| ≥ 1. GO and KEGG enrichment analyses were subsequently performed on the DEGs. The *k*-means clustering algorithm was employed to classify the aforementioned DEGs (Yang et al., 2022). For WGCNA, all expressed genes were used as a base gene set. WGCNA was performed via TBtools II (power = 12, minModuleSize = 30, and MEDissThres = 0.25) (Chen et al., 2023).

### 2.5 Identification of CBF-dependent and flavonoid biosynthesis-associated pathway genes in *H. moellendorffii*

The methods used to identify genes in CBF-dependent pathway and associated with flavonoid biosynthesis were similar to published methods (Liu et al., 2024a). Briefly, the protein sequences of reported flavonoid biosynthesis-associated genes and CBF-dependent pathway genes were downloaded from NCBI databases. These BLASTP searches were used to call putative protein sequences using the full-length protein sequences of *H. moellendorffii* as queries. Finally, putative proteins lacking the same Pfam domains (Mistry et al., 2021) as the query genes were excluded from the analysis.

### 2.6 Metabolomics assay and data analysis

After the leaf samples had been dried at -50°C, 50 mg of each powdered sample was extracted with 400 μL of extraction solvent (methanol:water = 4:1, v/v) containing an internal standard (L-2-chlorophenylalanine at 0.02 mg/mL). The sample mixture was ground for 6 minutes at -10°C and 50 Hz in a cryogenic tissue grinder, followed by ultrasonic extraction at 5°C and 40 kHz for 30 minutes. The samples were then allowed to stand at -20°C for 30 minutes and then centrifuged at 4°C and 13000 × g for 15 minutes; the resulting supernatant was transferred to a vial with an insert for instrumental analysis. The samples were analysed on a Thermo Fisher Scientific UHPLC-Q Exactive HF-X system (Shanghai Meiji Biopharma Technology Co., Ltd.). A 3-μL sample was separated on an HSS T3 column (100 mm × 2.1 mm i.d., 1.8 µm) and introduced into the mass spectrometer. Mobile phase A consisted of 95% water and 5% acetonitrile (with 0.1% formic acid), and mobile phase B consisted of 47.5% acetonitrile, 47.5% isopropanol, and 5% water (with 0.1% formic acid). The flow rate was set to 0.40 mL/min, and the column temperature was maintained at 40°C. Mass spectrometry signals were acquired in both positive and negative ionization modes, with a mass scan range of 70–1050 m/z. The sheath gas flow rate was 50 psi, the auxiliary gas flow rate was 13 psi, the auxiliary gas heater temperature was 425°C, the capillary temperature was 325°C, the positive mode ion spray voltage was set at 3500 V, and the negative mode ion spray voltage was set at -3500 V, with a normalized collision energy of 20–40–60 eV in a cyclic manner. The resolution for the first mass spectrometer was 60000, and that for the second mass spectrometer was 7500; data-dependent acquisition (DDA) mode was used.

The raw LC−MS data obtained in the analysis were imported into the metabolomics processing software Progenesis QI (Waters Corporation, Milford, MA, USA) for baseline filtering, peak identification, integration, retention time correction, and peak alignment, resulting in a data matrix of retention time, mass-to-charge ratio, and peak intensity. To obtain information on metabolites, the MS and MS/MS spectral information was matched against the public metabolite databases HMDB (http://www.hmdb.ca/) and Metlin (https://metlin.scripps.edu/) as well as against an in-house database built by Meiji. The data matrix was then preprocessed as follows: missing values were removed using the 80% rule, which retains variables with nonzero values in at least 80% of the samples, followed by imputation of missing values with the minimum value in the original matrix. To reduce errors caused by sample preparation and instrument instability, the response intensities of the MS peaks were normalized using the total sum normalization method, resulting in a normalized data matrix. Variables with relative standard deviation (RSD) >30% in the QC samples were removed, and the data were log10 transformed to obtain the final data matrix for subsequent analysis. The preprocessed data matrix was subjected to principal component analysis (PCA) and orthogonal partial least squares-discriminant analysis (OPLS-DA) via the R package ropls (version 1.6.2). The stability of the model was evaluated using cross-validation. Significantly different metabolites were selected on the basis of the variable importance in the projection (VIP) values obtained from the OPLS-DA model and the *P* values from Student’s t test, with VIP > 1 and *P* < 0.05 indicating significant differences. The differentially abundant metabolites were annotated using the KEGG database to identify the pathways in which they are involved. Pathway enrichment analysis was performed via the Python package scipy.stats, and significant biological pathways were identified through the use of Fisher’s exact test.

### 2.7 Cojoint analysis of the transcriptome and metabolome

The transcriptome and metabolome data obtained from the same three biological replicate samples were used for cojoint analysis. Correlation analysis of the WGCNA modules with flavonoid metabolites was performed on the MetWare cloud platform (https://cloud.metware.cn) (parameters: powerEstimate=18, mergeCutHeight=0.25, minModuleSize=50). The correlation between transcription factors and flavonoid biosynthesis structural genes was calculated via the rcorr function of the R language Hmisc software package (Harrell and Harrell, 2019), and a correlation clustering heatmap was plotted via the heatmap plugin in TBtools II (Chen et al., 2023).

### 2.8 Reverse-transcription quantitative PCR assay

To confirm the expression of flavonoid biosynthesis-related genes, a total of 19 genes were selected for RT−qPCR. These included two hub genes from WGCNA, four potential interacting TFs and 13 structural genes associated with the flavonoid biosynthesis pathway. The primers used were designed using Primer3 (https://bioinfo.ut.ee/primer3-0.4.0/) (**Supplementary Table S5**). The samples were subjected to RNA-seq. RT−qPCR was performed on a Roche Light Cycler 96 (Roche, Switzerland) using actin as an internal reference. The PCR system consisted of 10 µL of Power SYBR^®^ Green PCR Master Mix (Applied Biosystems, Foster City, CA, USA), 1 µL of forward primer (10 μM), 1 μL of reverse primer (10 µM), 1 µL of cDNA template, and 2 µL of nuclease-free H_2_O. The reaction conditions are described in Liu et al (Liu et al., 2018). The relative expression levels of the target genes were determined via the 2^-⊿⊿CT^ method (Livak and Schmittgen, 2001).

## 3 Results

### 3.1 Full-length sequencing and annotation

**Figure 1:**
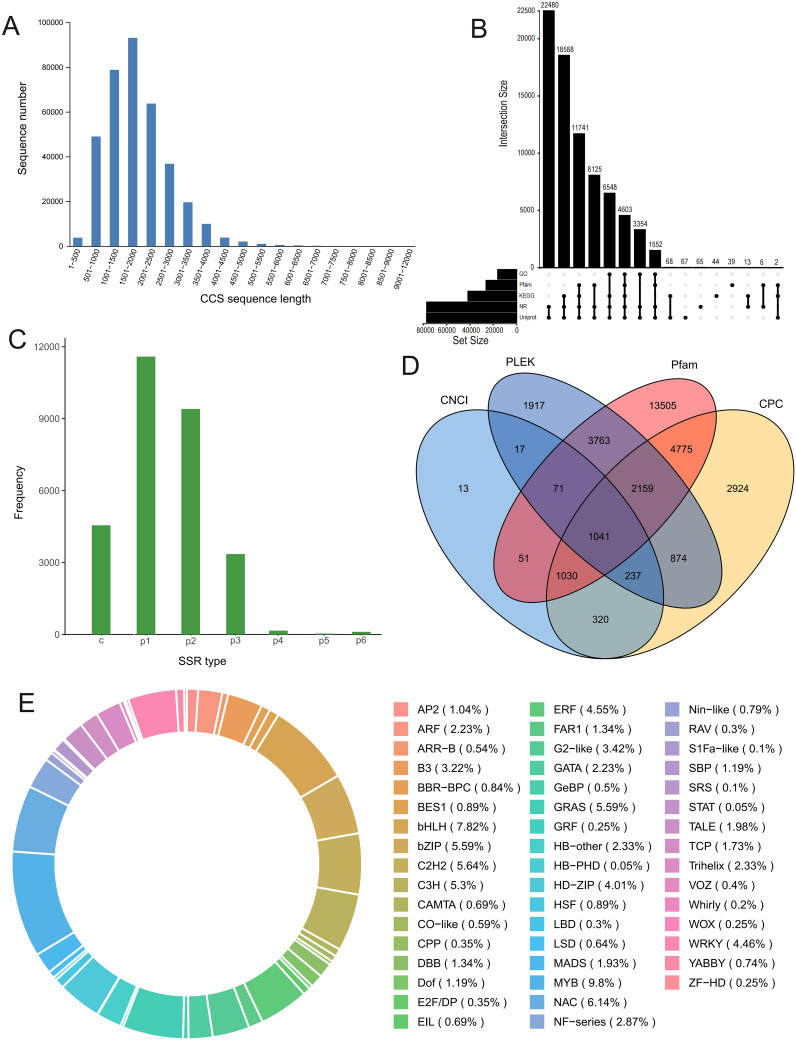
Full-length RNA sequencing analysis. **(A)** CCS (circular consensus sequencing) reads length distribution histogram. **(B)** Upset plot of the isoform numbers annotated by the five databases. **(C)** SSR (simple sequence repeats) type distribution histogram. **(D)** Venn plot of predicted lncRNA numbers using four different methods. **(E)** Types and ratio of predicted transcription factors (TFs).

Pooled full-length transcriptome sequencing yielded approximately 29 Gb of clean subreads with an average read length of 1755 bp. After data filtering, a total of 340,273 full-length nonchimeric (FLNC) CCS reads were obtained, with an N50 value of 2032 bp. These reads were primarily distributed in the 500 bp−4000 bp range, with the highest frequency in the 1501 bp−2000 bp interval (**Figure 1A**). After redundant reads were removed, a total of 82,673 high-quality isoforms were obtained. The BUSCO evaluation value was 83.9%, suggesting that the transcript reference library has potential value. The full-length transcript sequences were aligned with five databases (NR, UniProt, GO, KEGG, and Pfam), and 77,075 transcripts were found to be annotated in at least one of these databases. The majority of the isoforms (~93%) were annotated via either UniProt or NR (**Figure 1B**). Subsequently, 29,187 SSR loci, primarily of types p1 and p2, were identified (**Figure 1C**). We continued to predict potential long noncoding RNAs (lncRNAs) and ultimately identified 1041 potential lncRNAs that were supported by all four prediction results (**Figure 1D**). Given the crucial role of transcription factors (TFs) in stress responses, we also predicted TFs for this transcriptome library. A total of 2,020 TFs belonging to 49 classes were identified; those whose abundance represented more than 5% of the total were MYB (9.80%), bHLH (7.82%), NAC (6.14%), bZIP (5.59%), GRAS (5.59%), C2H2 (5.64%), and C3H (5.30%) (**Figure 1E**).

### 3.2 Gene family and phylogenetic evolution analysis

**Figure 2:**
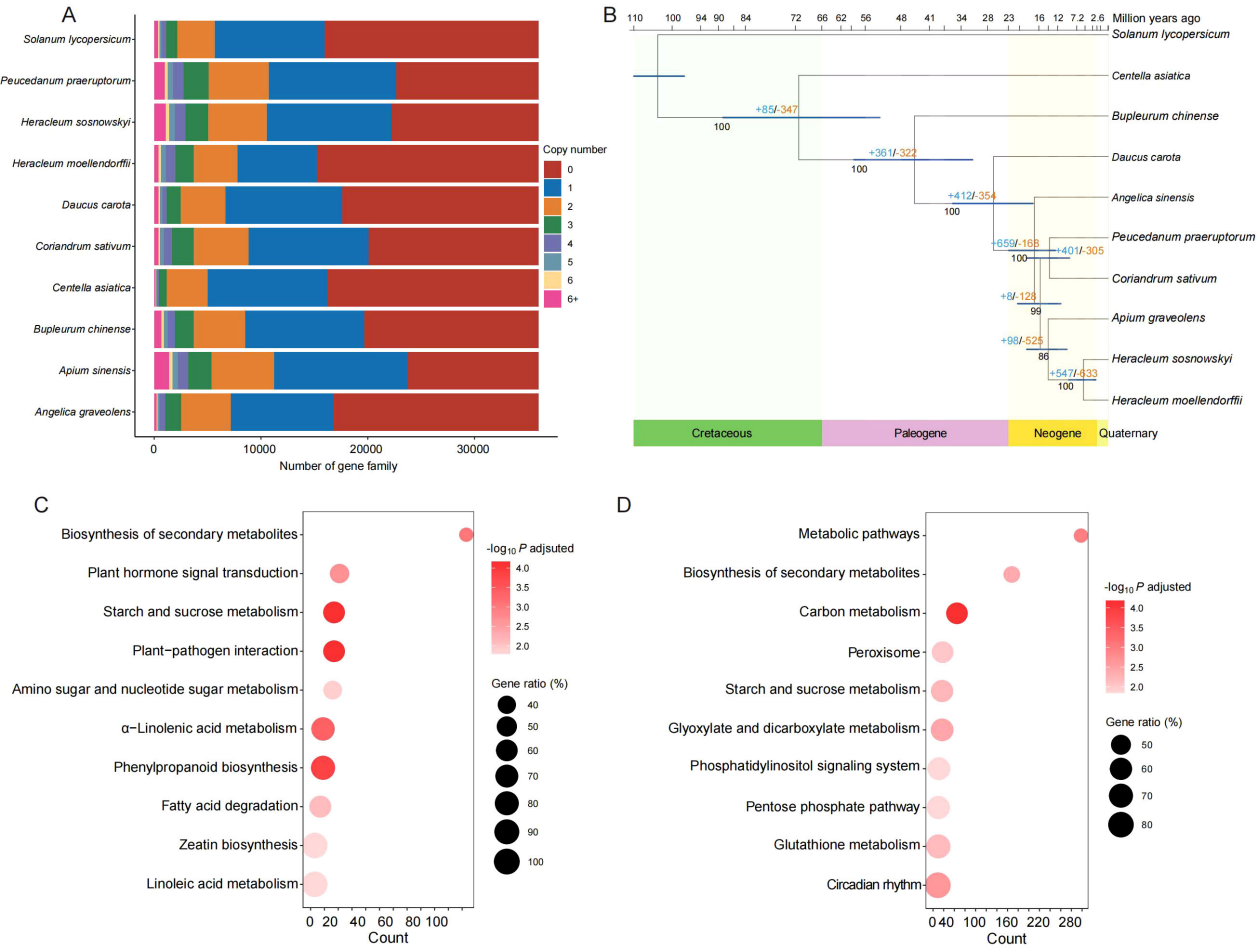
Comparative genomic analysis. **(A)** Bar plot of gene family component. **(B)** Phylogenetic tree and divergence time of the ten species. “+” and “-” indicate gene numbers being expanded and contracted, respectively. **(C)** KEGG enrichment analysis of the contracted genes in *H*. *moellendorffii*. **(D)** KEGG enrichment analysis of the expanded genes in *H*. *moellendorffii*.

Among the 82,673 isoforms obtained for *H. moellendorffii*, a total of 58,675 were predicted to encode proteins. Further gene family clustering analysis revealed 35,992 families; *H. moellendorffii* contained 15,238 of these families, and 1,210 of those were species-specific, encompassing 3,518 genes (**Figure 2A**; **Supplementary Figure S1C**). The species-specific gene families enriched in GO or KEGG terms were related to “fatty acid metabolic process”, “starch and sucrose metabolism”, and “biosynthesis of amino acids” (**Supplementary Figures S1A, B**). Among all the gene families, 581 single-copy orthologous genes were identified. These were used to construct a phylogenetic tree for nine species within the family Apiaceae and the outgroup *S. lycopersicum* (**Figure 2B**). The results indicated that *H. moellendorffii* is most closely related to *H. sosnowskyi*, with a divergence time of approximately 6 million years ago (Mya); its divergence occurred after the divergence of the common ancestor of *A. graveolens* and *H. moellendorffii* (~14 Mya). In the genome of *H. moellendorffii*, 547 genes underwent expansion, and 633 genes experienced contraction. The contracted genes were enriched in the biosynthesis of secondary metabolites, plant hormone signal transduction, and starch and sucrose metabolism (**Figure 2C**), whereas the expanded genes were enriched in metabolic pathways, the biosynthesis of secondary metabolites and carbon metabolism (**Figure 2D**).

### 3.3 Differentially expressed genes identified through RNA-seq

**Figure 3:**
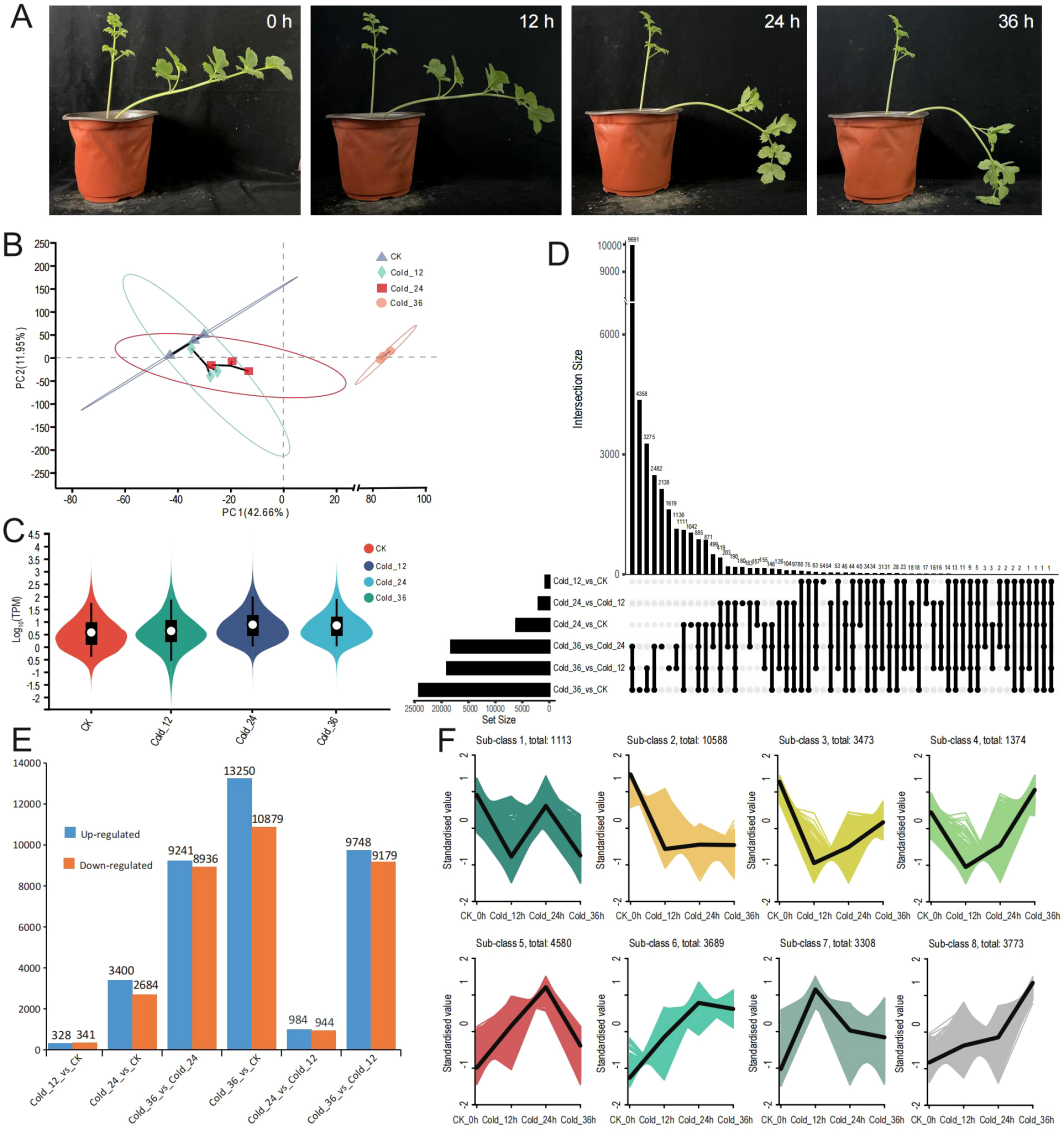
The sample appearance and RNAseq analysis. **(A)** The performance of *H*. *moellendorffii* at 0 h, 12 h, 24 h and 36 h after 4°C treatment. **(B)** The result of PCA (principal component analysis) using gene expression TPM (transcripts per million) values of samples at the four time points. **(C)** Violin plot of gene expression TPM (transcripts per million) values. Different colors represent different samples. **(D)** Six comparison groups’ upset plot of differentially expressed genes (DEGs). **(E)** Six comparison groups’ column diagram of up-regulated and down-regulated gene numbers. **(F)** Eight sub-class DEGs using *k*-means cluster method.

To further assess the performance of the full-length transcriptome library and investigate the mechanisms of the cold stress response in *H. moellendorffii*, we conducted transcriptome sequencing under cold stress conditions. As shown in **Figure 3A**, compared with those of the control at 0 h, the leaves of plants subjected to cold stress presented evident wilting, with increased severity as the duration of the treatment increased. Transcriptome sequencing generated a total of 78.37 Gb of clean data, with each sample exceeding 6.15 Gb and with a Q30 value greater than 96% (**Supplementary Table S1**). PCA revealed a high degree of similarity among the biological replicates (**Figure 3B**). Violin plots indicated that samples from the plants subjected to cold treatment for three different amounts of time (Cold_12, Cold_24, and Cold_36) presented higher gene expression levels than did the control CK, particularly Cold_24 and Cold_36 (**Figure 3C**). We performed DEG analysis using six different pairwise comparisons. In the comparisons between Cold_12 vs. CK, Cold_24 vs. CK, Cold_36 vs. CK, Cold_24 vs. Cold_12, Cold_36 vs. Cold_12 and Cold_36 vs. Cold_24, we identified 669 (**Supplementary Figure S2A**), 6084 (**Supplementary Figure S2B**), 24,129 (**Supplementary Figure S2C**),1928 (**Supplementary Figure S2D**), 18,927 (**Supplementary Figure S2E**), and 18,177 (**Supplementary Figure S2F**) DEGs, respectively. The shared and unique DEGs identified in the six comparisons are shown in **Figure 3D**, resulting only five commonly shared DEGs and 54, 1042, 4358, 180, 1619, 2138 unique DEGs in Cold_12 vs. CK, Cold_24 vs. CK, Cold_36 vs. CK, Cold_24 vs. Cold_12, Cold_36 vs. Cold_12 and Cold_36 vs. Cold_24, respectively. The number of DEGs increased progressively with time in the plants that were subjected to cold stress, indicating a progressively increased response to cold stress in *H. moellendorffii*. In all the pairwise comparisons, the number of upregulated genes exceeded the number of downregulated genes (**Figure 3E**). The numbers of upregulated genes in Cold_12 vs. CK and in Cold_24 vs. Cold_12 were 328 and 984 respectively, whereas the number of upregulated genes observed in the other pairwise comparisons was 3400 minimally in Cold_12 vs. CK, reaching a maximum of 13,250 in Cold_36 vs. CK. This suggests that a milder transcriptional response occurred at the first two time points (12 h and 24 h) and that a more intense response occurred at 36 h.

To further analyse the expression trends of different DEGs, we performed *K*-means clustering analysis on all DEGs across the comparison groups. This resulted in 8 subclasses, representing 8 distinct expression patterns (**Figure 3F**); KEGG enrichment analysis was conducted for each subclass gene set (**Supplementary Figure S3**). The top enriched pathways included “oxidative phosphorylation” (subclass 1), “photosynthesis” (subclass 2), “ribosome biogenesis in eukaryotes” (subclasses 4 and 6), “autophagy” (subclass 5), “protein processing in the endoplasmic reticulum” (subclasses 7 and 8), “flavonoid biosynthesis” (subclass 3), and the typical “plant hormone signal transduction” pathway (subclasses 6, 7, and 8). Notably, subclass 2, subclass 3, subclass 4, and subclass 6 enriched pathways are related to flavonoid biosynthesis, implying that flavonoid biosynthesis plays a significant role in the cold stress response of *H. moellendorffii*. Additionally, genes involved in the CBF-dependent pathway, including the CBF-type genes Hmo.29573, Hmo.121080, and Hmo.34904 were rapidly upregulated in response to cold stress, whereas BTF (Hmo.21414), BYBC1 (Hmo.114249), and PIF (Hmo.112347) were downregulated (**Supplementary Figure S4**).

### 3.4 Metabolomic analysis and identification of differentially accumulated metabolites

**Figure 4:**
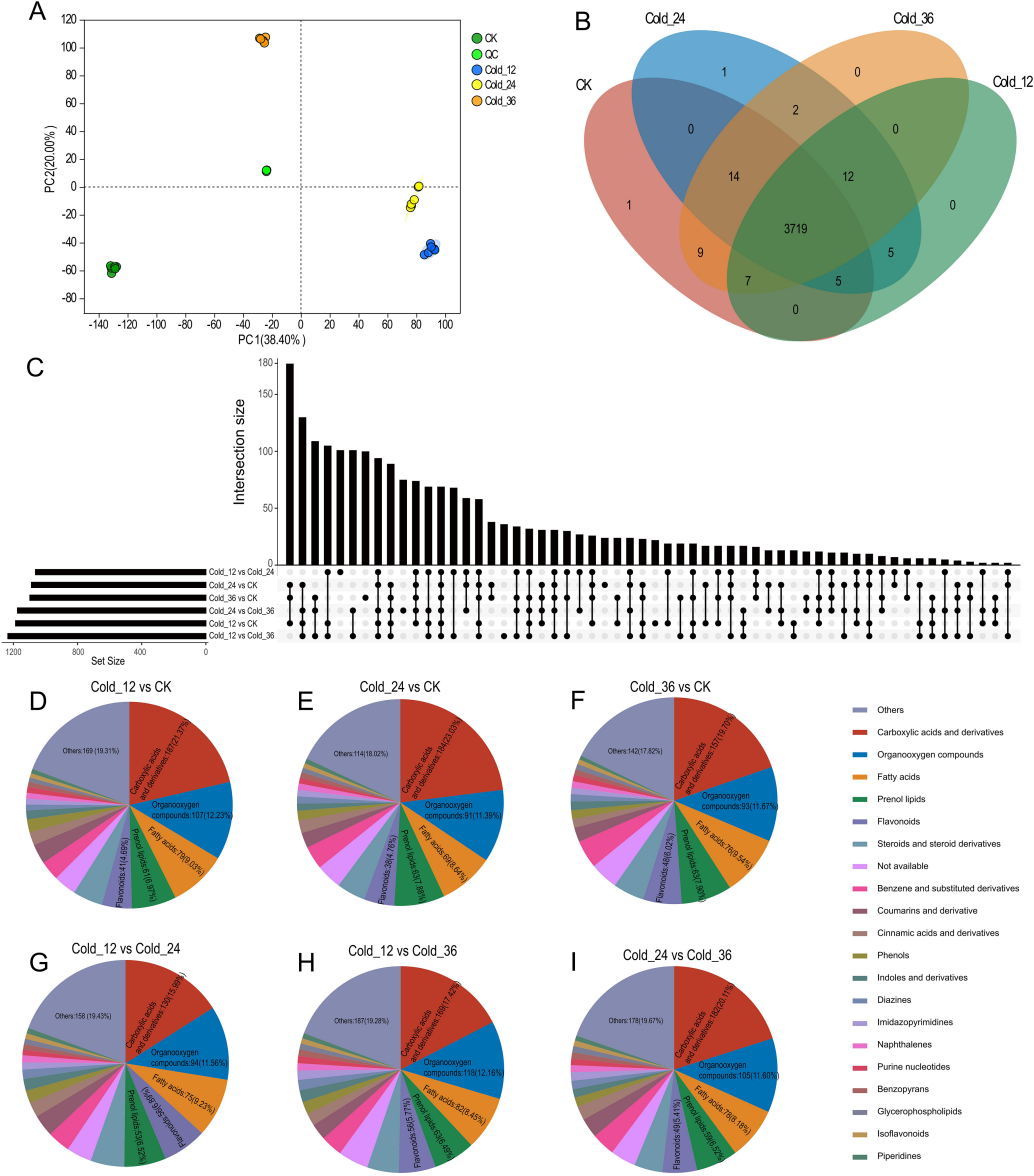
Metabolome analysis. **(A)** The result of PCA (principal component analysis) using metabolite content values of samples at the four time points. **(B)** Venn plot of annotated metabolites in the samples at four time points. **(C)** The differential expressed metabolites (DAMs) upset plot of the six comparison groups. **(D-I)** A pie chart categorizing DAMs in the corresponding six comparison groups. Top six categories were showed in the pie chart.

Using samples from the same time points as those used for RNA-seq, we also conducted metabolomic experiments. PCA revealed minor differences among the six biological replicates, whereas obvious differences were observed among the samples obtained at different time points (**Figure 4A**). The nontargeted metabolomic assay identified 3775 annotated metabolites, 3719 of which were detectable in samples from all four time points (**Figure 4B**). As in the transcriptome analysis, DAMs were identified for six pairwise comparisons: Cold_12 vs. CK, Cold_24 vs. Cold_12, Cold_24 vs. CK, Cold_36 vs. Cold_24, Cold_36 vs. Cold_12, and Cold_36 vs. CK; 1186, 1062, 1087, 1174, 1234, and 1097 DAMs, respectively, were identified, in these comparisons (**Figure 4C**). Excluding the category classified as “others”, the top 5 categories of DAMs in all the comparison groups were carboxylic acids and derivatives, organooxygen compounds, fatty acids, prenol lipids, and flavonoids (**Figures 4D**-**I**). Compared with those in CK, the DAMs in Cold_12, Cold_24, and Cold_36 were enriched predominantly in the following pathways: “nucleotide metabolism”, “flavonoid biosynthesis”, “pyrimidine metabolism”, and “purine metabolism”. Additionally, DAMs that differed among Cold_12, Cold_24, and Cold_36 were enriched in “flavonoid biosynthesis” (**Supplementary Figure S5**), indicating that flavonoids may be important metabolites in *H. moellendorffii*’s response to cold stress.

### 3.5 Correlations between modules identified by WGCNA and flavonoid metabolites

**Figure 5:**
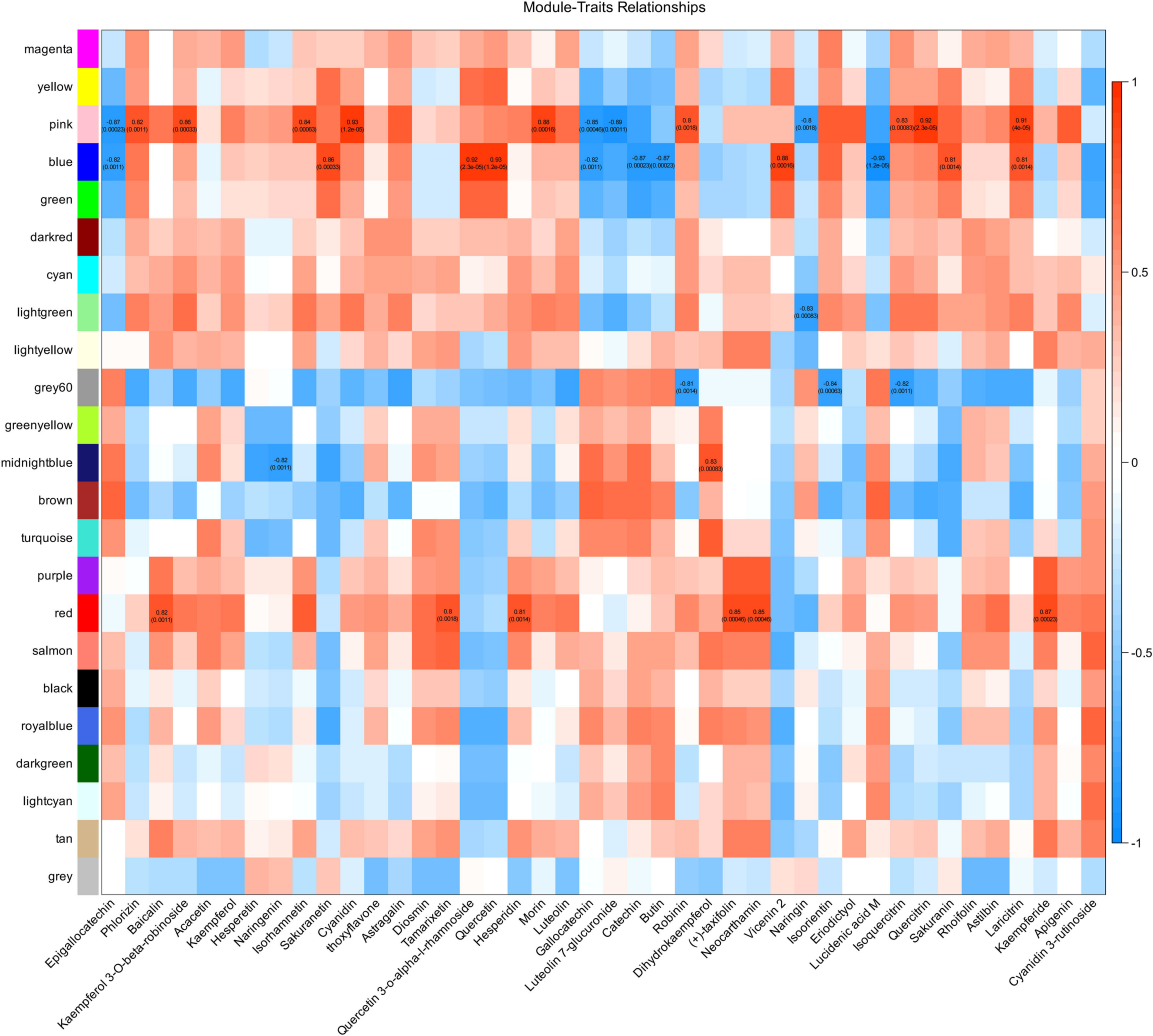
Correlation analysis between gene modules by weighted correlation network analysis (WGCNA) and flavonoid metabolites. The left different colors represent different modules. In each correlation block, the number at the top indicates the correlation values, and the corresponding *P* values are indicated in parentheses below.

To further explore the associations between gene expression and flavonoid metabolites, we conducted weighted gene coexpression network analysis (WGCNA) and calculated the correlations between expression modules and flavonoid metabolites. Co-expression module was detected by hierarchical cluster tree (**Supplementary Figure S6A**), and a total of 22 modules were identified through WGCNA (**Supplementary Figure S6B**); each module identified five potential hub genes (**Supplementary Table S2**). A total of 42 flavonoid metabolites were involved in the correlation analysis with the modules. There were 36 module–metabolite paired relationships with correlation coefficients greater than 0.8 or less than -0.8 with *P*<0.01 (14 negative correlations and 22 positive correlations), covering five modules (pink, blue, light green, gray60, midnight blue, and red) and 29 metabolites. Notably, the pink module was highly significantly positively or negatively correlated with 13 flavonoid-related metabolites, and the blue module was highly significantly positively or negatively correlated with 11 flavonoid-related metabolites, suggesting that genes in these two modules play important roles in flavonoid metabolism in *H. moellendorffii* under cold stress conditions. The highest positive correlation coefficient, 0.93, was found for the relation pairs pink & cyanidin (*P*=1.2e-05) and blue & quercetin (*P*=1.2e-05). The highest negative correlation coefficient, -0.93, was found for the relationship between blue and lucidenic acid M (*P*=1.2e-05) (**Figure 5**).

### 3.6 Dissection of the flavonoid metabolic pathway involved in cold stress

**Figure 6:**
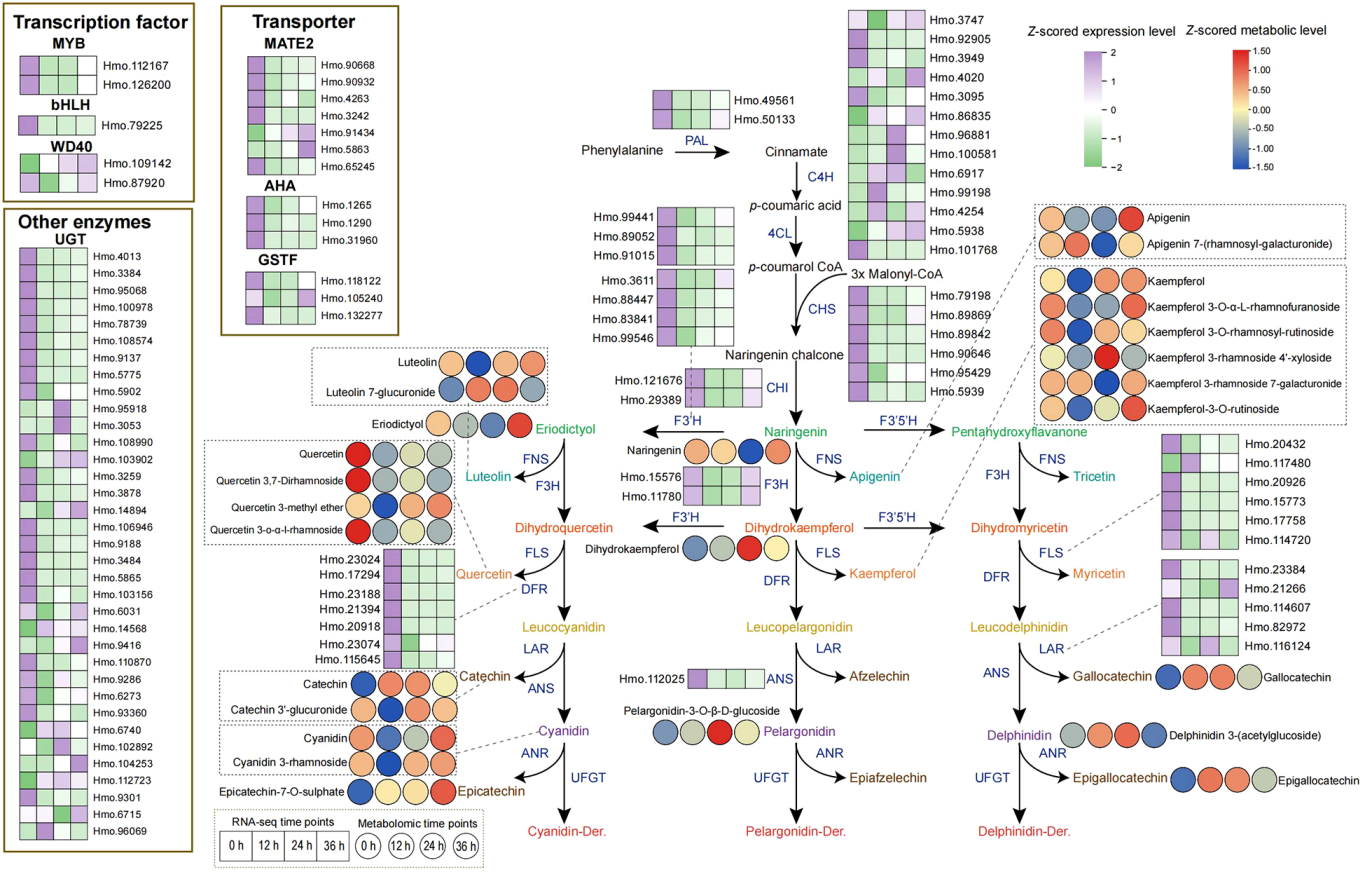
DEGs and DAMs of flavonoid biosynthesis pathway induced by cold stress. The solid arrows indicate direct reaction flows in the pathway. The enzymes encoded by the related DEGs in the flavonoid biosynthesis pathway are located next to the arrows or dashed lines. The left and right adjacent square or circular heat maps represent the corresponding DEG or DAM values (row *Z*-scored) at 0 h, 12 h, 24 h and 36 h after 4°C treatment, respectively, with TPM (transcripts per million) or content values.

The flavonoid biosynthetic pathway has been demonstrated to participate in the cold stress response of plants. Therefore, we utilized the isoforms from the full-length transcriptome to identify genes involved in the flavonoid biosynthesis pathway in *H. moellendorffii*. A total of 108 genes were identified; 90 of these encode constitutive enzymes, 5 encode transcription factors (TFs), and 13 encode transporters (**Supplementary Table S3**). We subsequently integrated the differentially expressed flavonoid biosynthesis genes and the differentially expressed flavonoid-related metabolites into a pathway diagram (**Figure 6**). The DEGs were found to include the structural genes PAL, C4H, 4CL, CHS, CHI, F3H, DFR, ANS, FLS, and LAR. Genes such as Hmo.49561 and Hmo.50133 (belonging to PAL), Hmo.121676 and Hmo.29389 (belonging to CHI), and Hmo.15576 and Hmo.11780 (belonging to F3H) showed decreased expression at 12 h, followed by sharply increased expression at 24 h and 36 h. The C4H genes Hmo.3747, Hmo.4020, and Hmo.96881, as well as the UGT-type genes Hmo.95918 and Hmo.3053, presented relatively high expression levels after 24 h and 36 h of treatment. Five transcription factors (including MYB, bHLH, and WD40) and 13 transporters were also found to be differentially expressed. Like the expression patterns of structural genes of the PAL, CHI, and F3H types, expression of the AHA-type transporter Hmo.1265 also tended to decrease under cold stress conditions, followed by a gradual increase. Metabolite accumulation also exhibited a variety of patterns. Gallocatechin, luteolin 7-glucuronide, and pelargonidin content rapidly increased after cold stress began and then decreased at 36 h but remained higher than in the control. Epicatechin-7-O-sulfate content increased after cold stress, and this compound accumulated gradually until reached a peak level at 36 h. The content of epigallocatechin, however, initially increased sharply under cold stress conditions and then gradually decreased. Overall, the accumulation of most flavonoid metabolites at 36 h of cold stress was greater than that in the control.

### 3.7 Interaction network between structural genes associated with flavonoid biosynthesis and TFs

**Figure 7:**
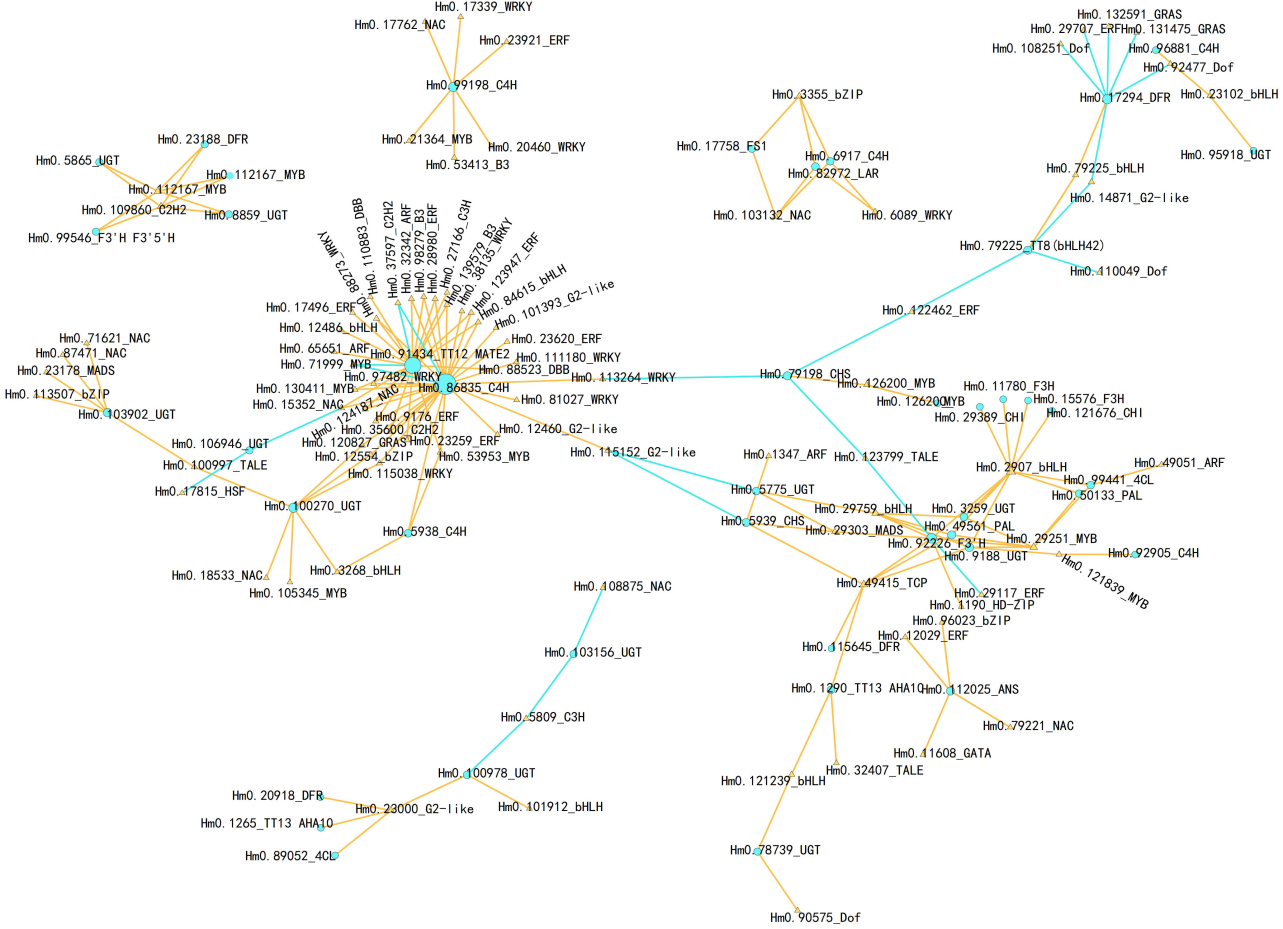
Connection network of structural genes in flavonoid biosynthesis pathway and TFs. The networks were visualized with Cytoscape software (version 3.9.1). The orange triangle indicates TFs, while the sky-blue circle indicates structural genes. The orange line indicates positive correlations, while the sky blue line indicates negative correlations.The Pearson’s correlation coefficient with r > 0.90 and *P* value ≤ 0.01 were maintained.

TFs that interact with structural genes are widely involved in the regulation of metabolite synthesis. Therefore, we conducted a correlation analysis between the differentially expressed TFs and DEGs in the flavonoid biosynthesis pathway and used the results to construct an interaction network (**Figure 7**; **Supplementary Table S4**). TFs that potentially interact with C4H (Hmo.86835) and TT12 MATE2 (Hmo.91434) were the most numerous (33 and 22 TFs, respectively), and most of these interactions were positive, suggesting that these interactions may serve as regulatory hubs in response to cold stress. Additionally, the structural genes Hmo.92226 (F3’H), Hmo.103902 (UGT), and Hmo.99198 (C4H) were significantly positively correlated with multiple TFs, including TFs belonging to the NAC, WRKY, and MYB types. In contrast, Hmo.17294 (DFR) was significantly negatively correlated with bHLH (Hmo.23102), GRAS (Hmo.132591, Hmo.131475), ERF (Hmo.29707), and Dof (Hmo.108251, Hmo.92477) expression. Importantly, a single TF can interact with multiple structural genes. For example, expression of the C2H2 TF Hm0.109860 was significantly correlated with expression of Hm0.112167 (MYB), Hm0.23188 (DFR), Hm0.5865 and Hm0.8859 (UGT), and Hm0.99546 (F3’5’H). The expression levels of two MYB-type TFs, Hm0.112167 and Hm0.29251, correlated significantly with those of four flavonoid-related genes (Hm0.23188, Hm0.5865, Hm0.8859, and Hm0.99546) and six flavonoid-related genes (Hm0.3259, Hm0.49561, Hm0.50133, Hm0.9188, Hm0.92226, and Hm0.99441), respectively. Hm0.29759 expression was correlated with that of three UGTs (Hm0.3259, Hm0.5775 and Hm0.9188), one PAL gene (Hm0.49561), and one F3’H gene (Hm0.92226). Hm0.49415 (TCP-type TF) expression was correlated with the expression of six different types of structural genes (Hm0.115645, Hm0.1290, Hm0.49561, Hm0.5939, Hm0.9188, and Hm0.92226).

### 3.8 Real-time quantitative PCR confirmation of the expression of flavonoid biosynthesis pathway genes

**Figure 8:**
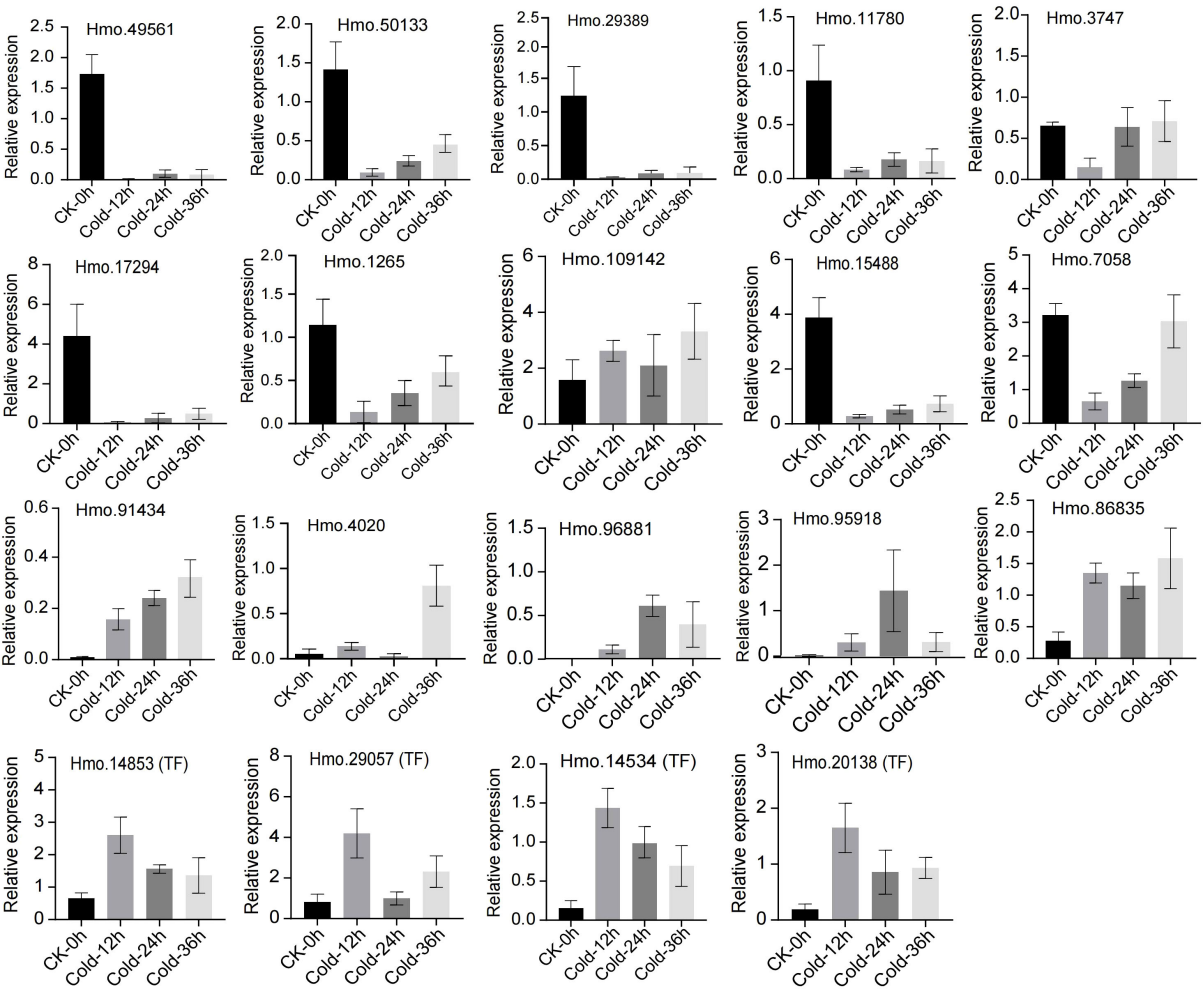
Real-time quantitative PCR (RT-qPCR) of 19 flavonoid biosynthesis related genes. Standard deviations calculated by three biological replicates are shown with error bars.

To confirm the expression of flavonoid biosynthesis pathway genes and their potential regulators, we selected 19 genes for RT−qPCR (**Figure 8**). The expression correlation (R^2^) between RNA-seq and RT-qPCR was 0.8954 (**Supplementary Figure S7**). The expression of most structural genes, including Hmo.49561 (PAL), Hmo.50133 (PAL), Hmo.29389 (CHI), Hmo.11780 (F3H), Hmo.3747 (C4H), Hmo.17294 (DFR) and Hmo.1265 (AHA), decreased sharply after 12 h of treatment and then increased with increasing exposure time until 36 h. Hmo.15488 and Hmo.7058 presented similar expression patterns. In contrast, the structural genes Hmo.4020 (C4H), Hmo.96881 (C4H), Hmo.86835 (C4H) and Hmo.95918 (UGT) were strongly upregulated after cold treatment, especially at 24 h and 36 h. Notably, Hmo.91434, which encodes a TT12 MATE2 transporter, showed consistently high and increased expression during the 36 h of treatment. All the four TFs presented obviously greater expression at all time points tested (12 h, 24 h, and 36 h) than they did in the CK samples. Most of the selected genes presented expression patterns similar to those indicated by the TPM values measured via RNA-seq.

## 4 Discussion

### 4.1 Construction of a full-length transcriptome isoform library of *H. moellendorffii* provides valuable information for the functional genomics study of this species

A reference genome can greatly accelerate functional genomic research (Wang and Han, 2022c). However, the genome of *H. moellendorffii*, a nonmodel species, remains uncharacterized, which greatly hindered its functional genomic research. In this study, PacBio-based full-length transcriptome sequencing analysis enabled to identify a panel of genome-wide transcriptional isoforms, of which the BUSCO completeness assessment value was 83.9%, slightly higher than that of the *Sesamum indicum* genome v2 version (81.78%) (Wang et al., 2022a) and comparable to that of *Aethionema arabicum* (85.4%) (Fernandez-Pozo et al., 2021). Subsequently, we mined candidate genes that respond to cold stress in flavonoid biosynthesis pathway, thereby establishing favourable conditions for the functional genomics study of *H. moellendorffii*. However, a complete reference genome of *H. moellendorffii* is still necessary for studies of its evolutionary and functional genomics in the era of T2T genomes (Gladman et al., 2023).

### 4.2 Complex transcriptional and metabolic responses to cold stress in *H. moellendorffii*

Transcriptional regulation, culminating in the production of various metabolic products, is a classic response process of plants to low-temperature stress (Mehrotra et al., 2020). Although studies of the causal relationship between transcriptional changes and metabolic products are limited, increasing evidence suggests that transcriptional stress responses can lead to the accumulation of specific metabolites, enabling plants to cope with the adverse environment caused by cold stress. Through time-course transcriptome sequencing and metabolome analysis of plants exposed to cold stress, we characterized the DEGs (**Figure 3**) and DAMs (**Figure 4**) of *H. moellendorffii*. The number of DEGs increased gradually over time, with 19 transcription factors differentially expressed at Cold_12h compared with CK, 189 at Cold_24h, and 784 at Cold_32h. The results suggest that TFs sequentially initiated the response of *H. moellendorffii* to cold stress. The clustered DEGs were enriched in classic stress response pathways, such as “plant hormone signal transduction” (**Supplementary Figure S3**) (Tian et al., 2022). Among these DEGs, we also observed upregulation and downregulation of genes encoding proteins that participate in the CBF-dependent pathway (**Supplementary Figure S4**) (Hwarari et al., 2022). However, the hub genes identified through WGCNA did not include genes related to the CBF pathway, implying that although the CBF pathway in *H. moellendorffii* responds to cold stress, genes that are not dependent on the CBF pathway may play a critical role. Similarly, the DAMs that are found in plants exposed to cold stress can be divided into several different categories, mainly carboxylic acids and derivatives, organooxygen compounds, and fatty acids (**Figures 4D–I**) (Xu et al., 2022). Cold stress induced DAMs (Cold_12 vs. CK, Cold_24 vs. CK, Cold_36 vs. CK) were also enriched in KEGG pathways such as “nucleotide metabolism” and “flavonoid biosynthesis”, consistent with previous reports (Sun et al., 2021; Song et al., 2024) (**Supplementary Figure S5**). Strong correlations were detected between different expression modules and flavonoid metabolites via WGCNA. In addition, the *H. moellendorffii* specific gene families enriched in “fatty acid metabolic process”, “starch and sucrose metabolism”, and “biosynthesis of amino acids” (**Supplementary Figure S1A, B**), which may play roles in cold stress adaptation (Yue et al., 2015; Golizadeh and Kumleh, 2019). These results highlight the complexity of the transcriptional and metabolic responses of *H. moellendorffii* to cold stress. The transcriptome sequencing and metabolome data reported in this study provide an opportunity to elucidate the regulatory mechanisms involved in the cold response of *H. moellendorffii*.

### 4.3 Flavonoid biosynthesis and its regulation by interactions with TFs

Structural genes can interact with TFs to regulate metabolic pathways, thereby increasing plant resistance to abiotic stress. The flavonoid pathway has been shown to be involved in various stress responses, including the response to cold stress (Song et al., 2024). However, TFs related to flavonoid biosynthesis in *H. moellendorffii* have not been identified. Moreover, the relationships among the TFs that regulate flavonoid accumulation have not been elucidated. The transcriptome and metabolome results obtained in this study both indicate that the flavonoid pathway plays a significant role in the response of *H. moellendorffii* to cold stress. We identified 108 DEGs (including MYB, bHLH, and WD40) and 26 DAMs in the flavonoid biosynthesis pathway (**Figure 6**). Flavonoid metabolism is regulated by TFs such as MYBs and WRKYs (Naik et al., 2022; Wang et al., 2022b; Zhao et al., 2024). Interestingly, we observed these TFs among the DEGs (**Figure 3**), and in the correlation network analysis, we identified 196 TF–structural gene interaction pairs (correlation coefficient > 0.9, *P* < 0.01); these included MYB (Hm0.112167, Hm0.29251), bHLH (Hm0.29759), and WRKY (Hmo.88273, Hmo.38135, and others), and all of them correlated significantly with structural genes of the flavonoid pathway (**Figure 7**). These TFs could be potential targets for genetic modification; their identification provides a foundation for future research on the transcriptional regulation of flavonoid metabolism in *H. moellendorffii*.

## 5 Conclusions

To investigate the cold response of *H. moellendorffii*, a species for which no reference genome is currently available, PacBio-based full-length transcriptome sequencing was performed, a transcript isoform library was established, the evolutionary status of *H. moellendorffii* was clarified, and multiomics analysis was conducted via RNA-seq and metabolome assays during four key stages of cold stress treatment. A high-quality transcript isoform library was successfully constructed. Multiomic analyses revealed that different transcriptomes and metabolite expression patterns are found in the leaves of *H. moellendorffii* after cold treatment. Subclasses of both DEGs and DAMs were enriched in the flavonoid biosynthetic pathway. WGCNA also revealed expression modules harbouring hub genes that are highly correlated with cold stress, and some of these modules are strongly correlated with flavonoid metabolites. The deciphering of the flavonoid biosynthetic pathway in *H. moellendorffii* identified differentially expressed structural genes and metabolites. The roles of TFs in the flavonoid biosynthesis process were identified, and several potential TFs associated with flavonoid-related genes were screened. These results provide a foundation for elucidation of the functional genome of *H. moellendorffii* and the regulatory mechanisms involved in flavonoid biosynthesis in that species.

## Data Availability

The datasets presented in this study can be found in online repositories. The names of the repository/repositories and accession number(s) can be found in the article/**Supplementary Material**.

## Supplementary material

Supplementary Figure 1 Gene family analysis. **(A)** KEGG enrichment analysis of *H. moellendorffii* specific family. **(B)** GO enrichment analysis of *H. moellendorffii* specific family. **(C)** Upset plot of the gene clusters using the ten species.

Supplementary Figure 2 Volcano plot of differentially expressed genes (DEGs) in the six comparison groups. **(A)** Cold_12 vs. CK; **(B)** Cold_24 vs. CK; **(C)** Cold_36 vs. CK; **(D)** Cold_24 vs. Cold_12; **(E)** Cold_36 vs. Cold_12; **(F)** Cold_36 vs. Cold_24.

Supplementary Figure 3 KEGG enrichment analysis of 8 sub-class differentially expressed genes (DEGs) by *k*-means analysis.

Supplementary Figure 4 Clustering heat map of CBF-depenent differentially expressed genes (DEGs).

Supplementary Figure 5 KEGG enrichment analysis of differentially accumulated metabolites (DAMs).

Supplementary Figure 6 Weighted correlation network analysis (WGCNA) analysis. **(A)** Hierarchical clustering tree (dendrogram) of all genes with TPM>1. **(B)** Sample dendrogram and module heatmap. Each row color corresponds to the modules. The right panel represents the minimum (blue color) and maximum (red color) correlation coefficient.

Supplementary Figure 7 Pearson’s correlation between RT−qPCR and RNA-seq expression quantification.
