# Supplementary figures and images for: Multiomic analysis reveals that the flavonoid biosynthesis pathway is associated with cold tolerance in Heracleum moellendorffii Hance
Source: Front Plant Sci. 2025 Mar 14;16:1544898. doi: 10.3389/fpls.2025.1544898 (PMC11949932; doi:10.3389/fpls.2025.1544898)

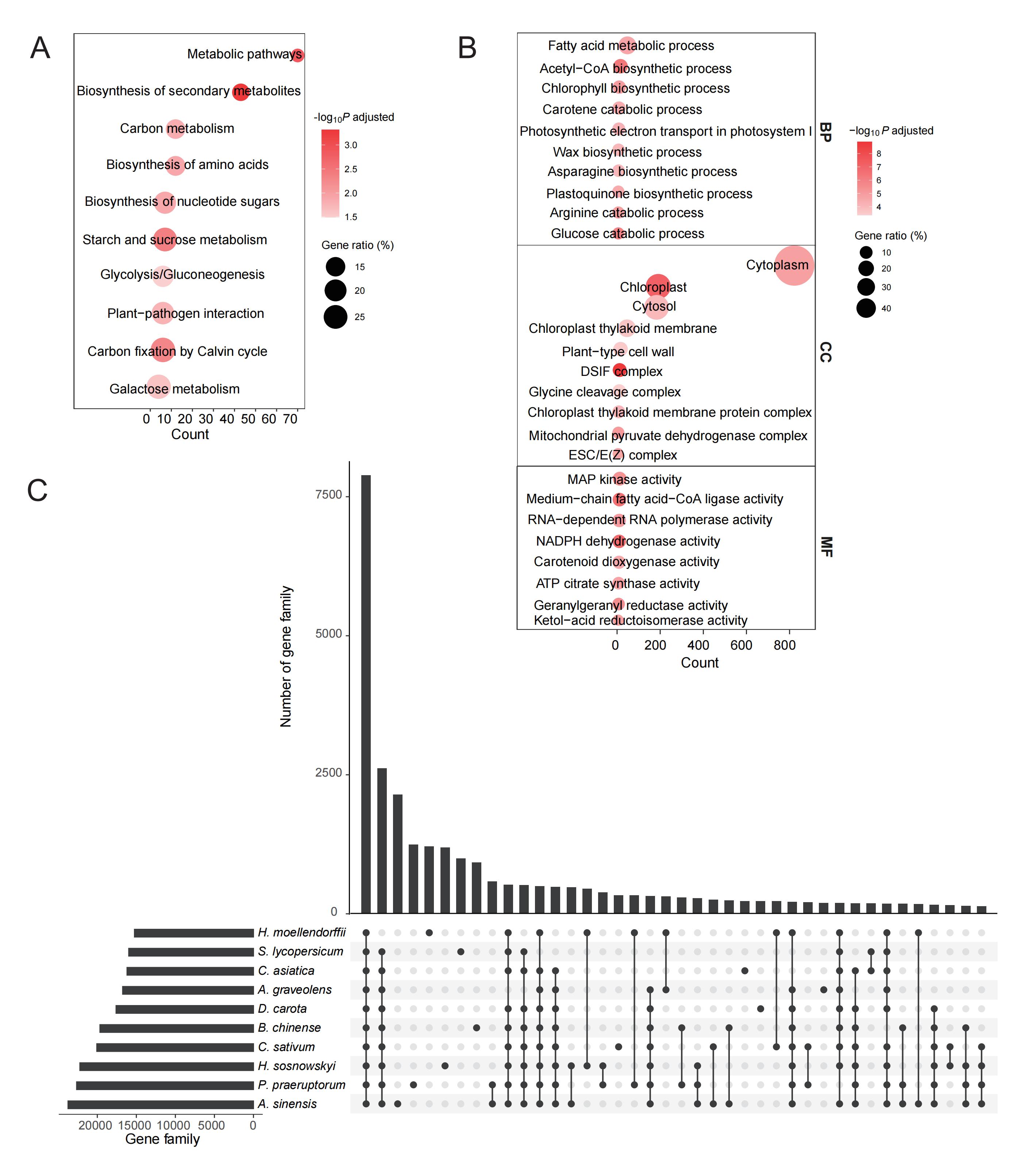

Supplement: Supplementary Figure 1 — Gene family analysis. (A) KEGG enrichment analysis of H. moellendorffii specific family. (B) GO enrichment analysis of H. moellendorffii specific family. (C) Upset plot of the gene clusters using the ten species. [file Image1.jpeg]

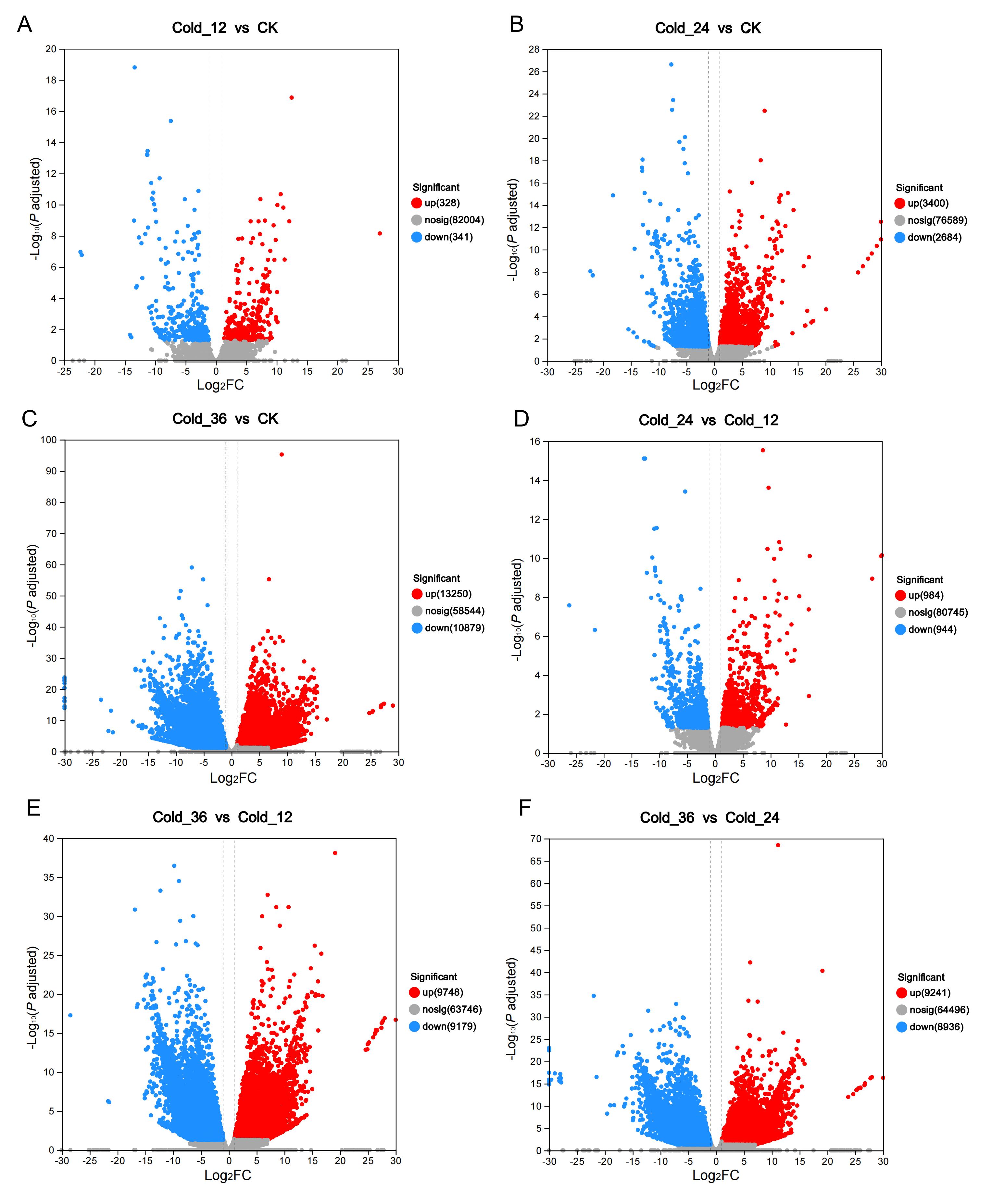

Supplement: Supplementary Figure 2 — Volcano plot of differentially expressed genes (DEGs) in the six comparison groups. (A) Cold_12 vs. CK; (B) Cold_24 vs. CK; (C) Cold_36 vs. CK; (D) Cold_24 vs. Cold_12; (E) Cold_36 vs. Cold_12; (F) Cold_36 vs. Cold_24. [file Image2.jpeg]

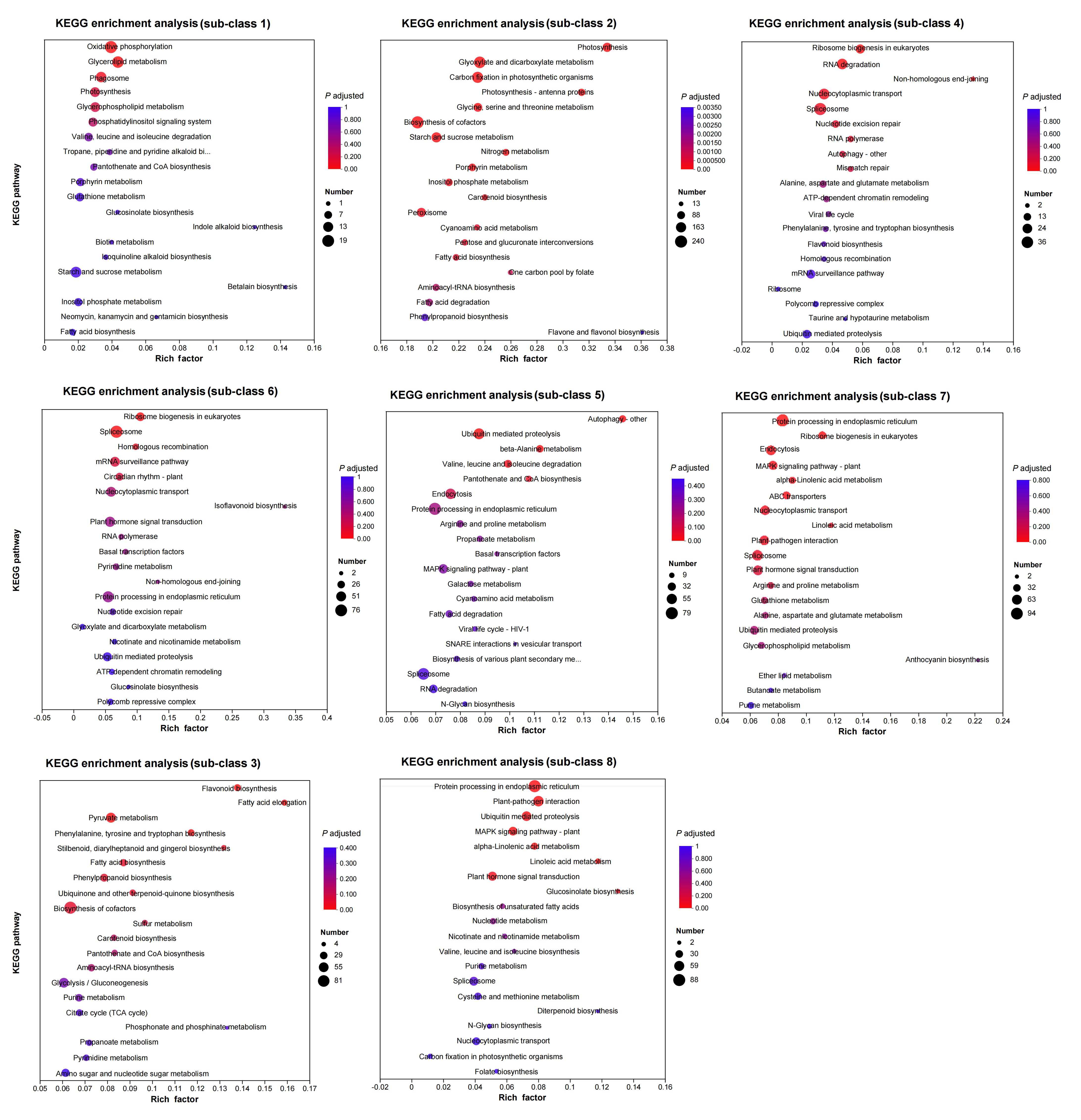

Supplement: Supplementary Figure 3 — KEGG enrichment analysis of 8 sub-class differentially expressed genes (DEGs) by k-means analysis. [file Image3.jpeg]

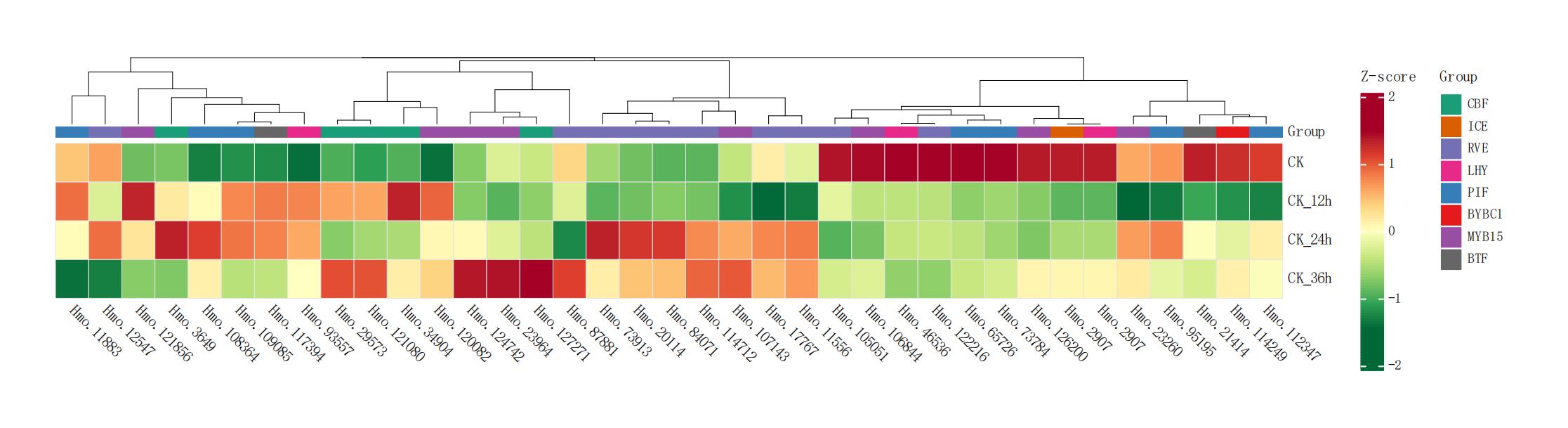

Supplement: Supplementary Figure 4 — Clustering heat map of CBF-depenent differentially expressed genes (DEGs). [file Image4.jpeg]

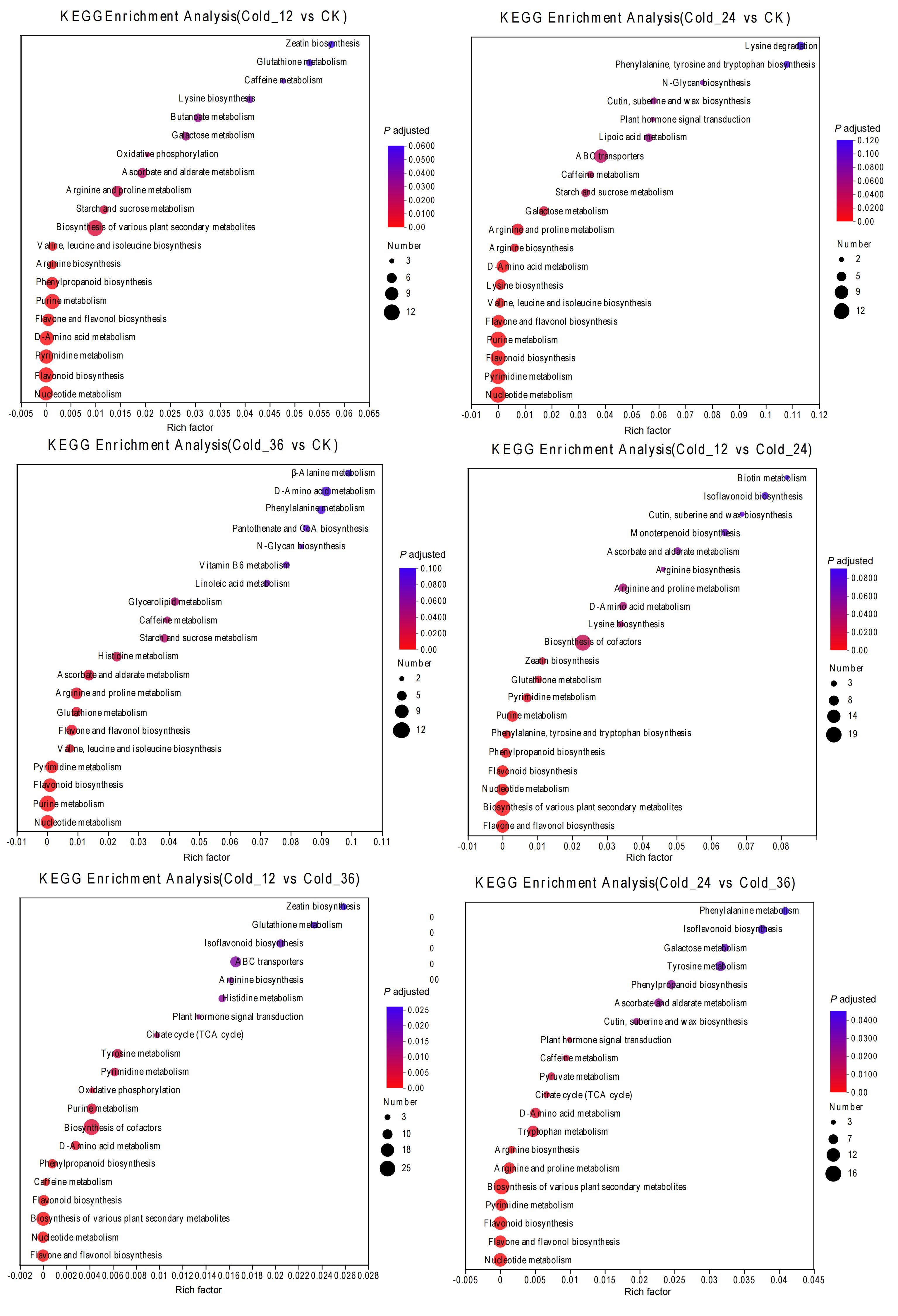

Supplement: Supplementary Figure 5 — KEGG enrichment analysis of differentially accumulated metabolites (DAMs). [file Image5.jpeg]

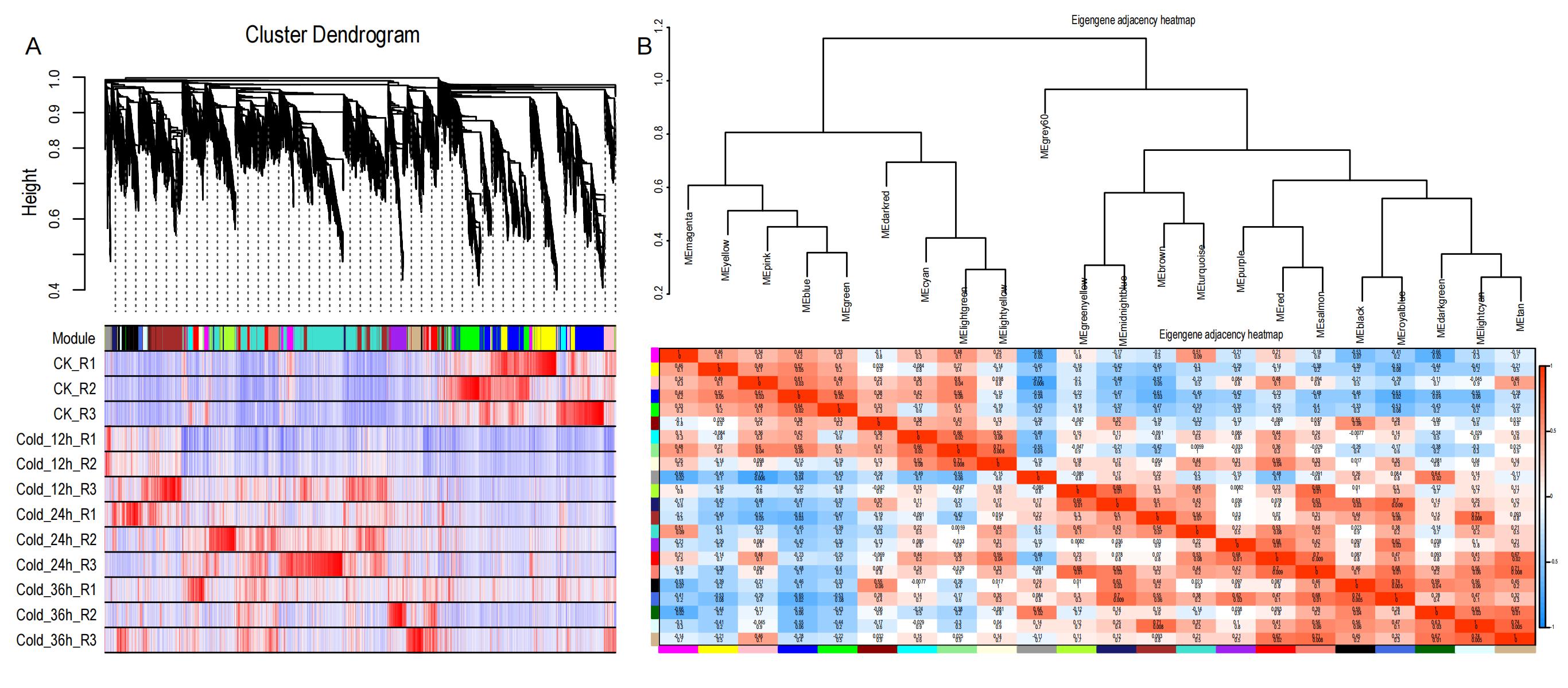

Supplement: Supplementary Figure 6 — Weighted correlation network analysis (WGCNA) analysis. (A) Hierarchical clustering tree (dendrogram) of all genes with TPM>1. (B) Sample dendrogram and module heatmap. Each row color corresponds to the modules. The right panel represents the minimum (blue color) and maximum (red color) correlation coefficient. [file Image6.jpeg]

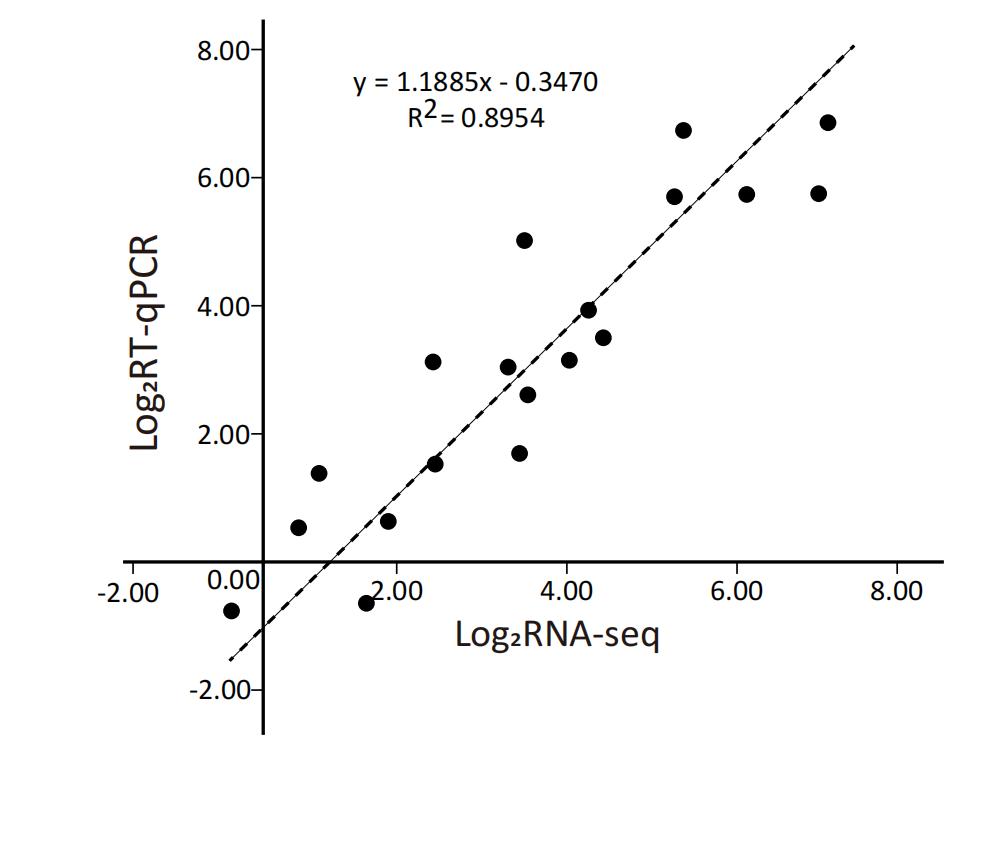

Supplement: Supplementary Figure 7 — Pearson’s correlation between RT−qPCR and RNA-seq expression quantification. [file Image7.jpeg]
